# Novel Molecules Targeting Metabolism and Mitochondrial Function in Cardiac Diseases

**DOI:** 10.2174/011573403X372565250331190001

**Published:** 2025-04-18

**Authors:** Samir Bolívar Gonzalez, César Vásquez Trincado, Karen Patricia Torres Rodríguez, Lizeth Paola Forero Acosta, María Fernanda Perez García, Steffy Saavedra-Castro, Sara Camila Castiblanco-Arroyave, Gerardo Manriquez Higuera, Luis Antonio Diaz-Ariza, Héctor Rodríguez Ortiz, Evelyn Mendoza-Torres

**Affiliations:** 1 Facultad de Química y Farmacia, Grupo de Investigación en Farmacia Asistencial y Farmacología, Universidad del Atlántico, Barranquilla, Colombia;; 2 Escuela de Química y Farmacia, Facultad de Medicina, Universidad Andrés Bello, Santiago, Chile;; 3 Faculty of Health, Exact and Natural Sciences, Universidad Libre Seccional Barranquilla, Barranquilla, Colombia;; 4 Grupo de investigaciones Biomédicas Uniremington, Corporación Universitaria Remington, Medellín, Colombia;; 5 Programa de Medicina, Universidad de Guadalajara, Guadalajara, México

**Keywords:** Cardiovascular disease, mitochondrial dynamics, heart failure, ischemia, cardiac hypertrophy, metformin, angiotensins, nicotinamide riboside

## Abstract

Cardiovascular diseases (CVD) are the leading cause of death worldwide, creating the need for new therapeutic strategies targeting the pathological processes involved. Mitochondria, which comprise one-third of cardiac cell volume, maybe a potential therapeutic target for CVD. Known primarily for energy production, mitochondria are also involved in other processes including intermediary metabolism, mitophagy, calcium homeostasis, and regulation of cell apoptosis. Mitochondrial function is closely linked to morphology, which is altered through mitochondrial dynamics, including processes such as fission and fusion, which ensure that the energy needs of the cell are met. Recent data indicate that mitochondrial dysfunction is involved in the pathophysiology of several CVDs, including cardiac hypertrophy, heart failure, ischemia/reperfusion injury, and cardiac fibrosis. Furthermore, mitochondrial dysfunction is associated with oxidative stress related to atherosclerosis, hypertension, and pulmonary hypertension. In this review, we first briefly present the physiological mechanisms of mitochondrial function in the heart and then summarize the current knowledge on the impact of mitochondrial dysfunction on CVD. And finally, we highlight the evidence from *in vitro*, *in vivo*, and clinical studies of the cardioprotective effects of drugs that preserve mitochondrial function in CVD. It is hoped that this review may provide new insights into the need to discover new pharmacological targets with direct actions on mitochondria that may provide combined therapeutic strategies to optimally treat these pathologies.

## 1 INTRODUCTION

Cardiovascular diseases (CVDs) have a great impact on the quality of life worldwide. These are a group of diseases of the heart and blood vessels that include coronary heart disease, cerebrovascular disease, rheumatic heart disease, and other conditions [1].

According to the WHO, CVDs are the leading cause of death globally, claiming an estimated 17.9 million lives each year [1]. The American Heart Association (AHA) annually reports statistics related to heart disease. According to NHANES data (National Health and Nutrition Examination Survey), CVD caused more deaths among individuals <85 years of age (596 786 *versus* 502 847 deaths) from 2017 to 2020 in comparison with cancer. Coronary heart disease (40.3%) was the leading cause of CVD death in the United States in 2021, followed by stroke (17.5%), other cardiovascular diseases causes combined (17.1%), high blood pressure (13.4%), heart failure (9.1%), and diseases of the arteries (2.6%) [2]. In the European region, CVDs cause more than 42.5% of all deaths annually, which represents 10,000 deaths every day, so there is no doubt that CVDs are the predominant cause of disability and premature death [3].

The economic cost of cardiovascular disease in the United States is estimated at $422.3 billion. The Global Burden of Cardiomyopathy based on 204 countries and territories in 2021: estimated 0.41 (95% UI, 0.38-0.44) million deaths from cardiomyopathy and myocarditis and an age-standardized mortality rate of 4. 95 (95% IU, 4.59-5.29) per 100,000. With respect to heart failure specifically, the total percentage of the population is projected to increase from 2.4% in 2012 to 3.0% in 2030. In fact, in 2019, the global burden in 204 countries and territories was found to be 56.2 (95% UI 46.4-67.8) million people living with left-sided heart failure [2].

Unique characteristics of the stroke burden in the Latin America and Caribbean region are high compared to global averages. There is an unfavorable temporal trend in the incidence of stroke in Latin America and the Caribbean for the younger population, particularly in young women [4].

In America, age-standardized CVD mortality rates decreased from 203.3 deaths per 100,000 inhabitants in 2000 to 137.2 deaths in 2019. When detailing the causes of mortality in women in the American Continent, ischemic heart disease is the main one, with a rate of 95.8 per 100,000 inhabitants. Other very important causes of death are: cerebrovascular accident with a rate of 50.3 per 100,000 inhabitants, hypertensive disease rate of 16.1 per 100,000 inhabitants, presenting a high burden of cardiovascular disease in women in the Region [5]. In the same region, an epidemiological transition is evident, where non-communicable diseases represented 77.4% of all deaths in 2000, reaching 80.7% in 2016. In women, non-communicable diseases occur in 85% and men in 77.1% [6]. The countries with the highest level of mortality from cardiovascular diseases are: Haiti, Guyana, Honduras, Suriname, and the Dominican Republic; being lower in Peru, Costa Rica, and Chile [4].

Due to the importance of these pathologies, we must know the important dynamics and processes in the function of the heart to be able to manage these diseases. It should be noted that mitochondria are an essential part of the cardiac function. These are the power plants of cells, maintaining their integrity through continuous cycles of biogenesis, fission, fusion, and degradation. In the cardiac system, dysregulation of mitochondrial dynamics has been shown to cause cardiac hypertrophy, heart failure, ischemia/reperfusion (I/R) injury, and various cardiac diseases, including metabolic and inherited cardiomyopathies. Furthermore, mitochondrial dysfunction associated with oxidative stress has been linked to atherosclerosis, hypertension, and pulmonary hypertension [7]. Mitochondrial fission and fusion are processes of the mitochondrial dynamics. The present findings demonstrate a novel and important concept that mitochondrial fission plays a critical role in Angiotensin II-induced hypertension and cardiovascular remodeling [8]. Knowing the impact of cardiovascular diseases and the great relevance of mitochondria in them, we seek to clarify, review, and carry out an exhaustive analysis of mitochondrial functions and new therapeutic strategies that target mitochondrial function. Therefore, this review aims to show new evidence that demonstrates the influential role of mitochondrial dysfunction in cardiovascular diseases and therapeutic strategies aimed at intervening in mitochondrial dysfunction.

## 2 MITOCHONDRIAL FUNCTION IN THE HEART

The heart provides blood flow to the systemic and pulmonary circulations [9]. Furthermore, histologically, the heart is composed of cardiomyocytes and other cells, which represent approximately 30% and 70% respectively [10]. 11 main cell types were found in samples taken from left-right ventricles, and left-right atria: atrial cardiomyocytes, ventricular cardiomyocytes, fibroblasts (FB), endothelial cells (EC), pericytes, smooth muscle cells (SMC), immune cells (myeloid and lymphoid), adipocytes, mesothelial cells and neuronal cells [11].

Cardiomyocytes are striated muscle cells found exclusively in cardiac muscle and have unique structures and properties that correlate with their contractile function. A physiological characteristic of cardiomyocytes is the intercalated discs, which contain cellular adhesions, such as gap junctions, to facilitate cell communication. These discs reduce internal resistance and allow action potentials to spread rapidly throughout the heart muscle through the passage of charged ions. Thus, the cardiac muscle acts as a functional syncytium with rapid synchronized contractions that are responsible for pumping blood throughout the body [12].

Non-cardiomyocytes partially consist of EC, fibroblasts, SMC, and immune cells [10]. Cardiac fibroblasts constitute just a group of mesenchymal cells that reside within the heart and are central players in normal cardiac physiology and cardiovascular diseases [13]. They are also the most common cell type represented in connective tissue and produce a diverse group of products including collagen types I, III, and IV, proteoglycans, fibronectin, laminins, glycosaminoglycans, metalloproteinases, and even prostaglandins [14]. Cardiac fibroblasts play essential roles in development by depositing collagens and other components of the extracellular matrix (ECM), in adult hearts, these mesenchymal cells constantly modify the microenvironment by degrading and depositing ECM. The fibroblasts are also responsible for cardiac fibrosis, which is the accumulation of ECM in response to a pathological stimulus [13].

During embryogenesis, the first organ formed is the heart. Mitochondria serve as the starter motor for contractile impulse in cardiomyocytes, thanks to ATP production, in addition to contributing to responses to hypoxia, oxidative stress, and biological functions such as regulating reactive oxygen species. The heart is one of the most active organs in the body producing approximately 30 kg of ATP per day. The adult mammalian cardiomyocytes contain about 6,000 mitochondria (30-40% of the total cell volume) [15].

### 2.1 Mitochondrial Function in Cardiomyocytes

Cardiomyocytes, the contractile units of the heart, are one of the most energy-consuming cell types, expending up to 30 kg of ATP per day [16]. Cardiomyocyte ATP pool comes mostly from mitochondrial oxidative metabolism (near 95%) [17], and slightly from glycolysis and TCA (tricarboxylic acid) cycle (as GTP) [18]. Cardiomyocyte energy production is integrated by metabolic processes such as fatty acid oxidation (β-oxidation), glycolysis, and TCA cycle; substrates like free fatty acids (FFAs), glucose, ketone bodies; enzymes involved in the metabolic pathways, and importantly, the mitochondrial electron transport chain (ETC) [19]. To cope with an appropriate and functional contraction, a healthy and well-perfused heart needs a constant energy supply from different sources. Specifically, around 70% of cardiac ATP is generated by fatty acid oxidation, and a minor proportion is derived from glucose and lactate oxidation [20, 21]. Importantly, mitochondrial fatty acid oxidation is one of the most important metabolic pathways in the heart and contributes up to 90% of acetyl-CoA produced in the cardiomyocyte oxidation [22] therefore, proper regulation of fatty acid uptake and metabolism is highly relevant for cardiac function.

In cardiomyocytes, the regulation of fatty acid and glucose metabolic pathways starts with the translocation of specific transporters to the sarcolemma, usually in response to insulin or increased cardiac contraction [23]. Glucose is primarily transported by the glucose transporter GLUT1, and under insulin- or contraction-stimulated conditions, it is also transported by GLUT4. After this, glucose is catabolized to pyruvate or stored as glycogen. On the other hand, long-chain fatty acids (LCFAs) uptake is mediated by CD36/FAT (fatty acid translocase) and FABP (fatty acid binding protein). Two main signaling pathways can lead to GLUT4 and CD36 exocytosis, under insulin action or contractile activity. Insulin receptor-dependent signaling activates phosphatidylinositol-3-kinase (PI3K), and consequently, the main metabolic regulator PKB/Akt and atypical PKC (PKC λ and ζ). Both PKB/Akt and atypical PKC, are necessary for GLUT4 translocation [24]. Akt-induced GLUT4 exocytosis is mediated by an inhibitory phosphorylation on AS160 (Akt substrate of 160 kDa) [25].

Insulin-dependent CD36 exocytosis is also dependent on PI3K and PKB/Akt activation [26]. On the other hand, elevated contractile activity temporarily increases AMP/ATP ratio which activates AMPK (AMP activated protein kinase), through LKB1 regulation [27]. Similar to Akt, AMPK induces AS160 phosphorylation [28]. Contractile activity mobilizes GLUT4 and CD36 from different endosomal storage compartments [23], to facilitate glucose and fatty acid uptake, respectively.

After the transport across the sarcolemma, LCFAs are processed by the enzyme acyl-CoA synthetase (ACS), located at the outer mitochondrial membrane (OMM). ACS converts LCFA to acyl-CoA, which can be esterified and stored in lipid droplets or subsequently transported to the mitochondrial matrix, through CPT proteins (carnitine palmitoyl-transferase 1 and 2). Finally, pyruvate and acyl-CoAs are oxidized in the mitochondrial matrix to generate ATP.

The adaptation of the mitochondrial network or content to different energetic demands represents a fundamental process to ensure the proper functioning of cardiomyocytes. The increase in mitochondrial mass and clearance of damaged mitochondria are key components of mitochondrial dynamics, which involve mitochondrial fusion, fission, biogenesis, and mitophagy [22, 29]. The mitochondrial biogenesis, the process where mitochondria grow and multiply [30], is necessary to maintain sufficient ATP production for cardiac contraction. Mitochondrial biogenesis involves the activation of PGC-1α (PPARγ coactivator-1α), and a consequent increase in NRF1 (nuclear respiratory factor 1) levels, which leads to an elevation of mitochondrial DNA and protein content. Thus, the activation of PGC-1α induces an increase in mitochondrial oxygen consumption and ATP production [30]. Additionally, modulation of the mitochondrial network cooperates to balance cardiomyocyte metabolism. In this context, insulin regulates mitochondrial function and fusion in cardiomyocytes through a mechanism that depends on increased levels of mitochondrial fusion protein OPA-1 [31].

Mitochondrial biogenesis, mitophagy, and fusion and fission balance play a key role in maintaining proper mitochondrial metabolism and quality control in the cardiomyocytes. In this context, an imbalance in the mitochondrial network dynamics, an alteration in mitophagy, and a reduction in mitochondrial biogenesis potentially lead to cardiomyocyte damage and death [32], which activates pathological processes in the heart and generates cardiac disease [33].

### 2.2 Mitochondrial Function in Cardiac Fibroblast

Cardiac fibroblasts (CF) are an important cell population of the myocardium, derived from progenitor cells of the epicardium, which after their epithelial to mesenchymal transition during gestation, colonize the cardiac interstitium providing structural support for the growing myocardium [34, 35]. CFs are the main source of extracellular matrix (ECM) production and they are part of one of the largest cell populations in the uninjured heart tissue [13], suggesting an important homeostatic role in maintaining the structural integrity of the ECM by constantly renewing, the extracellular matrix protein, collagen [36]. Similarly, CFs also respond to chemical and mechanical signals, since they express a variety of receptors that modulate cell proliferation/death, autophagy, cell adhesion, migration, expression of cytokines, chemokines, growth factors, and the differentiation to cardiac myofibroblasts (CMFs) [37]. The differentiation of CF to CMF is characterized by high expression of the protein α-SMA and excessive production of ECM proteins, as well as resistance to cell death, which results in their permanency in the injured heart, leading to maladaptive cardiac tissue remodeling [38].

**Fig. (1):**
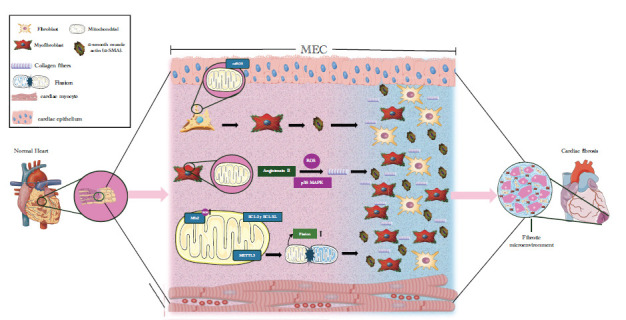
Myofibroblasts and cardiac fibrosis. Cardiac fibroblasts located in the ECM begin to differentiate into cardiac myofibroblasts through mitochondrial alterations, leading to the production of mtROS. Once differentiated, CMFs increase the expression of the α-SMA protein, thus aiding in their persistence and leading to cardiac tissue remodeling. Angiotensin II, which increases ROS production and p38-MAPK, thereby elevates collagen production and proliferation. At the mitochondrial level, anti-apoptotic proteins BCL-2 and BCL-XL are expressed, leading to a reduction in cell death; the elimination of mitofusin is activated, and ROS production increases; the METTL3 complex activates mitochondrial fission, causing the proliferation of FQs.

There are mitochondrial mechanisms of differentiation of CMFs [39] and profibrotic factors, such as adrenergic or angiotensin II (Ang II) stimulation, that increase the production of ROS and the activation/phosphorylation of p38-MAPK to increase proliferation and collagen production [40], through the negative regulation of superoxide dismutase 2 (SOD2) and catalase [41, 42]. On the other hand, considering that a characteristic feature of CMFs is the notable resistance to cell death, an action that contributes to their persistence in the stressful environment associated with fibrosis, one way in which mitochondria regulate apoptosis is through the release of apoptogen; indeed, it has been shown that fibrotic stress increases the expression of antiapoptotic proteins such as BCL-2 and BCL-XL, resulting in a decrease in cell death in models of cardiac fibrosis [43], Similarly, in CF, Ang II stimulation promotes apoptotic resistance by activating MAPK-ERK1/2 signaling to inhibit layerase-3 cleavage (Fig. **1**) [44].

In addition to the accepted functions of CMFs, recent studies suggest that mitochondrial abnormalities associated with metabolic remodeling determine the differentiation of CFs into CMFs. For example, alterations in mitochondrial morphology [45] and mtROS production [46] are reported to occur early in CMF differentiation. Likewise, it was shown that differences in the susceptibility of cardiomyocytes and CF to changes in mitochondrial function determine cell fate under the same pathological stimuli and in which the STAT3 protein plays a fundamental role [47]. On the other hand, they have shown that, during CFs activation, the removal of mitofusin (Mfn2), a key protein of the outer mitochondrial membrane, increased the production of reactive oxygen species (ROS), while the antioxidant, N-acetyl-l-cysteine (NAC), could attenuate the effect caused by the deletion of Mfn2. These data suggested that inhibition of Mfn2 could promote the activation of CFs by activating the endoplasmic reticulum-like protein kinase R kinase/activating transcription factor 4 (PERK/ATF4) signaling pathway and increasing the generation of ROS [48]. Similarly, other research highlights a novel mechanism where increased expression of methyltransferase complex 3 (METTL3) causes excessive mitochondrial fission, resulting in CF proliferation and migration leading to cardiac fibrosis [49]. Likewise, mitochondrial division inhibitor 1 (Mdivi-1) plays a beneficial role in cardiac fibrosis attenuating HR activation, collagen production, and fibrosis, by reducing the expression levels of dynamin-related protein 1 (DRP1) and abnormal mitochondrial fission of CFs in the border area of an infarct [50]. All these results suggest that changes in mitochondrial dynamics control CMF differentiation, in addition to the possible role of mitochondria-derived ROS in intracellular signaling in regulating fibrotic processes in cardiac tissue.

## 3 MITOCHONDRIAL DYSFUNCTION IN CARDIAC DISEASES

Heart diseases can be accompanied by alterations in mitochondrial function. Cardiac ischemia/reperfusion, heart failure, and cardiac hypertrophy are discussed in this section.

### 3.1 Cardiac Ischemia-reperfusion

**Fig. (2):**
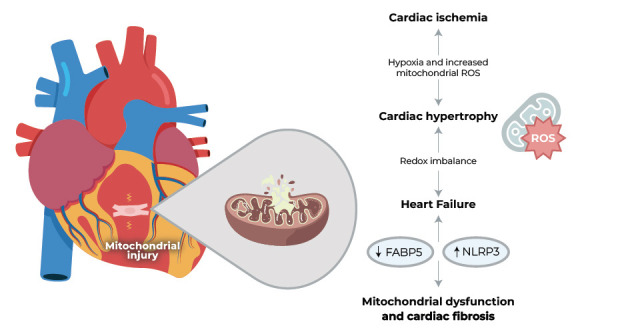
Consequences and mechanism of mitochondrial injury in the heart. **Abbreviations:** FABP5: fatty acid binding protein 5. NLRP3: nucleotide-binding domain, leucine-rich-containing family, pyrin domain-containing-3.

Ischemia is a state of hypoperfusion in tissues, attributed to different causes, which consequently leads to tissue injury [51]. Although the extent of damage will depend on the duration of ischemia [52], recent studies have established that additional damage is induced during the restoration of blood flow, this phenomenon is called ischemia-reperfusion injury [53] which is characterized by altered metabolic processes that are mainly attributed to mitochondria because they release ROS [54]. The increase of intracellular ROS during ischemia leads to the activation of cell death through an intrinsic pathway that is mediated by mitochondria [55]. This process is accompanied by the activation of proteins that modify the integrity of the mitochondrial membrane [51] mainly causing the opening of the mitochondrial permeability transition pore (mPTP) causing mitoptosis (Fig. **2**) [52].

During the reperfusion process, the reintroduction of oxygen into previously ischemic tissue generates excessive production of ROS in mitochondria, overcoming cellular antioxidant capacity and exacerbating oxidative damage. This oxidative stress affects various biomolecules, including lipids, proteins, and mitochondrial DNA, which compromises mitochondrial function and contributes to progressive cellular deterioration [56]. Furthermore, ROS accumulation activates inflammatory signals that increase immune cell infiltration to the affected area, prolonging cardiac damage and dysfunction. These inflammatory and oxidative processes converge to worsen the prognosis after an ischemia-reperfusion event [57].

Sustained opening of the mitochondrial permeability transition pore (mPTP) is a critical factor in mitochondrial dysfunction during reperfusion. This prolonged opening of the mPTP allows the release of proapoptotic factors and triggers the loss of mitochondrial membrane potential, precipitating programmed cell death events, such as necroptosis and apoptosis [58]. Disruption of mitochondrial homeostasis results in the selective elimination of dysfunctional mitochondria through mitophagy; however, in cases of extensive damage, this mechanism is not sufficient to restore cell viability [59]. Emerging therapeutic strategies seeking to limit reperfusion damage include mPTP inhibitors and mitophagy modulators, aimed at preserving mitochondrial integrity and improving cardiac recovery after ischemic events [60].

### 3.2 Heart Failure

Heart failure (HF) is characterized by dysfunction due to decreased heart capacity to send enough blood to the body to meet its needs [61]. HF involves signs and symptoms produced by evident structural and/or functional cardiac anomalies compatible with the presence of diastolic dysfunction/elevated left ventricular (LV) filling pressures, including elevated natriuretic peptides [62]. HF is mainly classified according to whether it presents with reduction (≤40%), slightly reduction (41-49%), or preservation (≥50%) of LV ejection fraction (LVEF) [63]. Ventricular failure is characterized by an energy deficit with a fundamental mitochondrial basis [64]. The primary cause of HF is due to energy metabolic changes such as decreased oxidation of mitochondrial pyruvate and an alteration in the pyruvate-lactate axis [65], generating high oxidative stress [66] and activation of cellular autophagy [15].

Some mitochondrial regulators such as TFAM (Mitochondrial transcription factor A) are essential for normal cardiac function. In *in vitro* studies, inactivation of TFAM in embryonic cardiomyocytes was lethal and mainly triggered by the elevation of ROS, DNA damage, and severely suppressed cardiomyocyte proliferation [67]. There are genes associated with mitochondrial alteration that would lead to congenital HF. The GTPases Mitofusin 1 (MFN1) and MFN2 act on the outer mitochondrial membrane to promote fusion. Genetic alteration of MFN1/2 at the cardiac level leads to mitochondrial fragmentation and fatal dilated cardiomyopathy [68]. Mitochondrial dysfunction has been widely linked to the development of HF. Alterations in mitochondrial metabolism lead to an inability to generate the energy necessary for the contractile function of the heart. HF is accompanied by redox imbalance, ROS generation, and impaired mitochondrial Ca^2+^ homeostasis [17].

### 3.3 Cardiac Hypertrophy

Hypertrophic cardiomyopathy is a mostly genetic disorder, where there are autosomal dominant mutations that phenotypically alter the sarcomere [69], which leads to an increase in the thickness of the myocardial wall, mainly the left ventricle [70] the main clinical manifestations that have been described are chest pain and dyspnea, palpitations, abnormal ECG [71].

Mitochondrial dysfunction is directly involved in cardiac hypertrophy due to the energy requirements of cardiac contraction, reflecting alterations in the mitochondrial membrane potential, increasing the amount of ROS, and leading to energy deficiency in the cardiomyocyte [72, 73]. A key aspect in the development of cardiac hypertrophy is the activation of the renin-angiotensin-aldosterone system (RAAS), where angiotensin II (Ang-II) plays a central role. Ang-II, through its binding to AT1R receptors, stimulates intracellular signaling pathways that promote cardiomyocyte hypertrophy. This process involves an increase in ROS production and mitochondrial dysfunction, exacerbating oxidative stress and contributing to pathological cardiac remodeling. Furthermore, Ang-II induces alterations in calcium homeostasis and mitochondrial biogenesis, which reinforces the progression toward energy dysfunction in the hypertrophied heart. Cardiac hypertrophy is followed by a process of cardiac remodeling accompanied by modifications in mitochondrial metabolism [74].

Hypertrophic cardiomyopathy is a disorder that is mostly genetic, where there are autosomal dominant mutations that phenotypically alter the sarcomere [69], which leads to an increase in the thickness of the myocardial wall, mainly the left ventricle [70]. The main clinical manifestations that have been described are chest pain and dyspnea, palpitations, and abnormal ECG [71]. Mitochondrial dysfunction is directly involved in cardiac hypertrophy due to the energy requirements of cardiac contraction, reflecting alterations in the mitochondrial membrane potential, increasing the amount of ROS, and leading to energy deficiency in the cardiomyocyte [72, 73].

### 3.4 Cardiac Fibrosis

In the search to establish whether there is a relationship between mitochondrial dysfunction and the development of cardiac fibrosis, different studies have been carried out that intrinsically demonstrate this correlation. One study demonstrated that the epigenetic reprogramming obtained in mitochondrial redox signaling leads to persistent metabolic changes in resident Right Ventricular fibroblasts (RVfib) that stimulate their rate of proliferation and collagen production [75], thus leading to the development of cardiac fibrosis. Moreover, mitochondrial fission has been revealed to regulate proliferation and collagen production in RVfib in any heart disease [76].

A study developed by Gao *et al.* 2021*.,* showed that the decrease in fatty acid binding protein 5 (FABP5) exacerbates hypertrophy and cardiac dysfunction induced by transverse aortic constriction (TAC), by exacerbating mitochondrial damage. Thus, FABP5 is significantly involved in fatty acid (FA) uptake, cytoplasmic transport, and FA oxidation, all of which are involved in the maintenance and regulation of mitochondrial structure and function. FABP5 deficiency in CFs was also found to lead to inappropriate mitochondrial ROS generation, impaired mitochondrial respiration, and CMF activation, which consequently plays an important role in the onset and development of cardiac fibrosis [77].

**Fig. (3):**
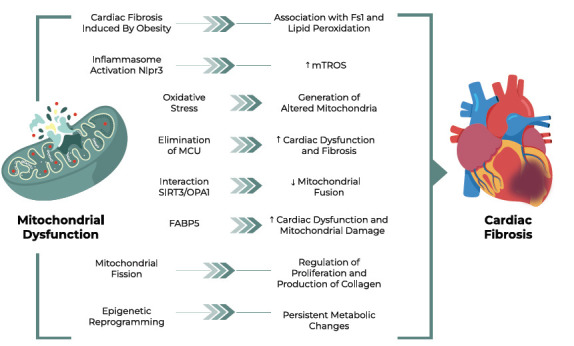
Mitochondrial dysfunction and cardiac fibrosis.

Similarly, it has been reported that within the mitochondrial dynamics in CF, sirtuin 3 (SIRT3) interacts directly with optic atrophy protein 1 (OPA1), producing its activation by deacetylation, therefore, CFs SIRT3/ KO manifest reduced mitochondrial fusion and loss of mitochondrial membrane potential uncoupling [29]. Another study found that deletion of the mitochondrial calcium uniporter (MCU) in adult HF exacerbates cardiac dysfunction, fibrosis, and CMF formation following MI and chronic angiotensin II (Ang II) administration (Fig. **3**) [78].

On the other hand, other studies have shown that the development of cardiac fibrosis is related to mitochondrial dysfunction, mainly due to the appearance of oxidative stress as a trigger for myocardial fibrosis and the high generation of altered mitochondria [47, 79].

**Fig. (4):**
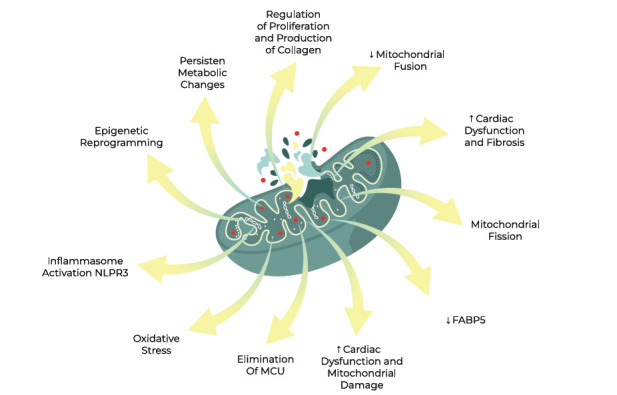
Events during mitochondrial dysfunction that influence cardiac diseases.

Likewise, NLRP3 inflammasome activation is involved in Ang II-induced cardiac fibrosis and hypertrophy, ultimately causing cardiomyopathy, likewise, Chen *et al.* 2021*.,* suggested that NLRP3 inflammasome-induced mitochondrial dysfunction it is implicated in Ang II-induced cardiomyopathy [80, 81]. Likewise, the role of mitochondrial dysfunction in the activation of the NLRP3 inflammasome has been studied. Shimada *et al.* 2012*.,* proposed that the signal II of the NLRP3 inflammasome induces mitochondrial dysfunction and a substantial increase in mtROS, which results in the release of mtDNA, oxidized in the cytoplasm where it binds to NLRP3, this cascade of events leads to the activation of the NLRP3 inflammasome and the generation of other stimuli [82, 83]. The possible activation of NLRP3 by mtROS has also been reported when there is mitochondrial damage [84] (Fig. **4**).

It is important to mention that the contribution of mitochondrial fission in the development of obesity-induced cardiac fibrosis has been suggested. A study in obese pigs demonstrated the association of mitochondrial parameters with cardiac fibrosis between the fission protein (Fis1) and lipid peroxidation, these were positively related to the content of cardiac collagen in the animals studied. Chen *et al.* 2020*.,* pointed out the direct correlation between cardiac oxidative stress, impaired mitochondrial biogenesis, mitochondrial quality control, and induced mitophagy, due to the consumption of a high-fat diet (HFD), therefore, it was suggested that mitochondrial variables contribute to the pathological mechanism of cardiac fibrosis [85].

Therefore, several factors can favor the development of cardiac fibrosis from mitochondrial dysfunction, such as epigenetic reprogramming in mitochondrial redox signaling, decreased FABP5 in CF, activation of the NLRP3 inflammasome, and the suggestive involvement of mitochondrial fission in the development of cardiac fibrosis.

## 4 NOVEL MOLECULES TARGETING METABOLISM AND MITOCHONDRIAL FUNCTION WITH THERAPEUTIC POTENTIAL FOR CARDIAC DISEASES

### 4.1 Metformin (MET)

Considering the significant role of mitochondrial function, especially energy production, in cardiac pathophysiology, the development of new therapeutic approaches focused on mitochondrial metabolism is highly relevant. One available drug which modulates mitochondrial energy production is metformin, a type 2 diabetes mellitus (T2D) drug from the biguanide class [86]. Metformin blocks mitochondrial complex I, reducing respiratory chain oxygen consumption [87], and therefore, decreasing ATP production. The ensuing increase in AMP/ATP ratio, activates AMPK (AMP-activated kinase), a major regulator of cardiac metabolism [88]. MET-induced AMPK activation has been related to a cardioprotective effect in ischemia/reperfusion (I/R) [89]. In this context, metformin modulated the inflammatory response during hypoxia/reoxygenation, attenuating JNK (c-Jun N-terminal kinase) activation, and consequently, NF-κB-induced transcription of pro-inflammatory cytokines, such as TNF-α and IL-6. Additionally, metformin-induced AMPK activation prevented the increase in ROS levels during hypoxia/reoxygenation [89].

Some studies have revealed the cardioprotective effect of MET through mitochondrial dynamics. MET has been recognized for its positive effects against cardiac I/R by decreasing mitochondrial fission, apoptosis, arrhythmias, and expansion of infarct size, in addition to preserving left ventricular function [90]. The effects of MET on apoptosis and mitochondrial fission suggest its potential positive effect on regulating the excessive growth of cardiac cells, characteristic of cardiac hypertrophy [91]. Likewise, a recent *in vitro* study demonstrated that MET exerts a protective effect against I/R injury in the retina through mitochondrial fusion, alleviating atrophy and cell loss induced by this process. MET acts by positively regulating the mitochondrial fusion proteins Mitofusin 2 (Mfn2) and Optic Atrophy (OPA1), which preserves mitochondrial morphology and function. Furthermore, MET reduces the production of reactive oxygen species (ROS) and protects against alteration of mitochondrial membrane potentials (MMP). This protective effect of metformin is mediated by the activation of the AMPK and its influence on PGC-1α signaling [92]. These findings support the consideration of MET as a promising therapeutic option in cardiac diseases through the regulation of mitochondrial dynamics and oxidative metabolism. Table **1** shows the effects of MET on the regulation of mitochondrial function in several studies.

On the other hand, previous clinical studies have suggested that MET may have positive effects on heart failure, but the evidence is still limited, and randomized trials are needed to support these findings. In this context, clinical results [93] suggest that the increase in myocardial efficiency during metformin treatment is not mediated by improvements in insulin action in patients with heart failure without diabetes. However, to date, no results have been reported. Likewise, the clinical trial NCT01690091 aims to evaluate the effect of MET on myocardial function, insulin resistance, and selected metabolic markers in patients with type 2 diabetes and heart failure in a randomized, placebo-controlled, crossover trial. No results have been published so far [94] (Table **2**).

It is expected that the results of preclinical studies will demonstrate that MET can improve myocardial function, insulin resistance, and other selected metabolic markers, which would confirm a positive effect on metabolism and mitochondrial dynamics. Future findings in clinical trials are expected to support the hypothesis that MET could have direct or indirect beneficial effects on myocardial efficiency, contractile function, and prevention of cardiac hypertrophy, through the regulation of mitochondrial dynamics and oxidative metabolism. Overall, MET continues to be the subject of active research and its role in the management of heart disease is being evaluated in ongoing clinical trials.

### 4.2 Resveratrol (RES)

Another molecule linked to mitochondrial function is resveratrol (RES). This well-known antioxidant agent has beneficial properties against cardiac pathological conditions such as heart failure (HF) or I/R-induced cardiac injury [95]. RES is a polyphenolic compound, present in various plant sources, such as grapes, berries, red wine, and peanuts [96]. This is a small molecule that exists in two structural isomeric forms, known as cis-resveratrol and trans-resveratrol. Most of the reported health benefits are associated with the latter variant [97]. RES exhibits anti-cancer, anti-aging, anti-inflammatory, neuroprotective, hepatoprotective, cardioprotective, antidiabetic, and antioxidant properties [98, 99]. The abundant presence of RES in red wine may explain why, despite the high prevalence of a high-fat diet in the French population, a relatively low incidence of cardiovascular diseases has been recorded in this population; this is known as the French paradox [100]. RES stimulates the activity of various proteins and enzymes in the body, such as AMPK, sirtuin 1 (SIRT1), superoxide dismutase (SOD), nuclear factor erythroid 2 related with factor 2 (NRF2), vascular endothelial growth factor (VEGF) and endothelial nitric oxide synthase (eNOS). RES also exerts inhibitory effects on cyclooxygenases (COX), phosphodiesterases (PDE), nuclear factor kappa B (NF-κB), aryl hydrocarbon receptor (AhR), phosphoinositide 3-kinase (PI3K), the mammalian target of rapamycin (mTOR), and the ribosomal protein S6K [96, 101-104] (Table **1**).

Research surrounding the effects of RES on heart disease encompasses a wide range of approaches in preclinical studies. A study conducted on mice and cell lines showed that RES exerts a protective effect on myocardial ischemia/reperfusion (I/R) injury through the modulation of AMPK/SIRT1- signaling pathways of the transcription factor Forkhead Box O1 (FOXO1) and the regulation of autophagy and oxidative stress. Furthermore, in this study, RES increased myocardial energy metabolism and reduced excessive ROS production in cardiomyocytes by activating the AMPK/SIRT1 signaling pathway, and effectively improving the level of autophagy [105]. Resveratrol treatment prevented ejection fraction decrease in a rat model of coronary artery ligation and reduced circulating levels of BNP. Resveratrol increased sirtuin 1 (SIRT1) protein levels and AMPK activation [95], suggesting that AMPK-dependent regulation of the cardiac metabolism, plays an important role in resveratrol cardioprotective effects.

RES can also significantly improve mitochondrial function in cardiomyocytes damaged by hypoxia/reoxygenation. It was observed that RES increases the content of MMP, ATP (Adenosine triphosphate) and SOD activity, while reducing the content of malondialdehyde (MDA). This protective effect of RES is attributed to its ability to promote mitochondrial fission and fusion through activation of the Sirt/Sirt3-FoxO signaling pathway. Furthermore, RES positively regulates the mRNA levels of key regulators of Sirt1/Sirt3-mediated mitochondrial fission and fusion [30, 101].

RES is effective in protecting mitochondrial function and structural integrity in cardiac tissue subjected to stress. ATP levels, indicative of mitochondrial metabolism, were increased with resveratrol pretreatment, suggesting an improvement in cellular energy function. Furthermore, a significant reduction in structural alterations of mitochondria was observed in tissues treated with RES, supporting its role in preserving mitochondrial dynamics and mitigating damage [31, 103].

Studies in progress focus on the need to evaluate the clinical efficacy of RES in cardiovascular diseases. For example, a clinical trial (NCT03525379) [104], aims to evaluate the effect of RES on metabolic function and skeletal muscle in men aged 50 to 75 years who have been diagnosed with heart failure with reduced ejection fraction (HFrEF) or heart failure with preserved ejection fraction (HFpEF). So far, no results have been reported (Table **2**).

Another clinical study conducted on patients with systolic heart failure showed a decrease in levels of cardiac biomarkers and inflammatory cytokines after resveratrol supplementation, suggesting a moderation of inflammatory processes. In addition, improvements in systolic and diastolic left ventricular function and global longitudinal tension were observed [105]. In summary, resveratrol treatment could have positive effects on mitochondrial dynamics and oxidative metabolism in patients with systolic heart failure.

The research focused on the effects of RES on cardiac diseases has provided a comprehensive perspective through various preclinical approaches. Research conducted on mice models and cell lines supports the potential value of RES as a therapeutic tool to address various cardiac conditions, pointing towards its ability to modulate critical cellular pathways associated with mitochondrial dynamics and oxidative metabolism, in search of improving cardiovascular health. In general, the RES continues to be an object of interest and study in the scientific field. Importantly, while these preclinical findings are encouraging, more research and clinical trials are still needed to confirm and fully understand the effects of RES in regulating mitochondrial dynamics and oxidative metabolism in heart disease.

### 4.3 Nicotinamide Riboside (NR)

On the other hand, nicotinamide riboside (NR) is a NAD+ (nicotinamide adenine dinucleotide) precursor that has been associated with several beneficial properties. In the cell, NR is transformed into nicotinamide mononucleotide by nicotinamide riboside kinase, boosting the biosynthetic pathway of NAD+ [106]. Unlike other NAD+ precursors, NR is considered favorable due to its safety and efficacy profile, without causing serious side effects or redness compared to other NAD+ precursors [107]. This vitamin B3 is bioavailable orally in humans and has shown potential benefits in protection against various pathological conditions like neurodegenerative diseases, diabetes, and hearing loss [108]. The synthesis of NR has been studied in yeast and mammalian cells, where it has been observed that it can be produced through the dephosphorylation of nucleotides such as nicotinamide mononucleotide (NMN) and nicotinic acid mononucleotide (NaMN). The uptake of NR into cells is carried out by equilibrating nucleoside carrier proteins, and once inside the cell, it can be phosphorylated to become NMN or deribosylated to subsequently transform into nicotinamide (NAM) [108, 109]. NR shows great potential in the medical field due to its protective effects and its role as a key precursor in the synthesis of NAD+, an essential coenzyme in various cellular processes [110].

In the constant search for therapeutic advances to address cardiac diseases through metabolism and mitochondrial dynamics, studies investigating the effects of NR have yielded promising results for its pharmacological potential. In a study conducted on mice C57BL/6N, was found that the oral administration of NR for 8 weeks improves NAD+ deficiency and reverses hyperacetylation of enzymes such as very long-chain acyl-CoA dehydrogenase (VLCAD) in failing hearts with preserved ejection fraction (HFpEF). This positive effect on NAD+ levels and mitochondrial function suggests that NR could have a therapeutic role in improving cardiac function in the context of HFpEF (Table **1**) [111].

In HF, NR-induced increase in NAD+ bioavailability has been linked to a decrease in ROS production, by protecting mitochondrial respiration, in PBMC (peripheral blood mononuclear cells), which was associated with a significant decrease of the pro-inflammatory cytokines [112]. As a NAD+-dependent protein deacetylase, SIRT3, an isoform of sirtuins located at the mitochondrial level, has been linked to the balance in energy metabolism and ROS production [113]. Certainly, SIRT3 overexpression reduced mPTP activity in cardiomyocytes with severe mitochondrial dysfunction [114].

Also, it has been shown that oral administration of self-assembled nanocrystal microspheres with NR and resveratrol (RES) (NR/RESms) for 8 hours not only significantly increased NAD+ levels in serum and various organs of mice, improving oral bioavailability of NAD+ but also showed notable efficacy in reducing myocardial infarct size in a mouse cardiac ischemia/reperfusion injury model, it should be noted that NR as a dietary supplement can be converted into NAD+ in cells to support mitochondrial energy metabolism (Table **1**) [115].

The results of this study provide strong evidence for the potential benefit of using NR as a strategy to strengthen NAD+ levels to mitigate the effects of pressure overload-induced cardiac hypertrophy. Furthermore, on the other hand, a specific mechanism has been explained that involves the decrease in oxidative stress and the inhibition of the activation of the NLRP3 inflammasome through the Sirtuin3-MnSOD signaling pathway (Table **1**) [116]. These findings support the notion that NR presents great potential as a new therapeutic intervention to treat cardiac diseases through metabolism and mitochondrial dynamics.

Likewise, clinical studies on NRs have emerged as an area of great interest and promise, with researchers directing their attention toward the potential impact of NRs on the prevention and treatment of heart disease, taking advantage of their ability to influence on metabolic pathways and key cellular mechanisms. In the pilot study NCT03727646 [112], the authors suggest that systemic inflammation in patients with heart failure (HF) is causally related to the mitochondrial function of peripheral blood mononuclear cells (PBMCs). Increasing NAD+ levels may have the potential to improve mitochondrial respiration and attenuate proinflammatory activation of PBMCs in HF.

Another clinical trial (NCT04528004) [117] aims to evaluate the mechanisms through which high levels of NAD+ in blood and myocardium in humans mediate changes in mitochondrial function, epigenetic and protein modifications, as well as inflammation. So far, no results have been reported, the study is in the recruitment process, it is estimated that the study will be completed in 2025 (Table **2**).

NR shows promising potential in the prevention and treatment of heart diseases, thanks to its influence on mitochondrial function, NAD+ levels, and mitigation of oxidative stress and inflammation. Results from preclinical studies are expected to demonstrate that nicotinamide riboside supplementation can improve cardiac function, reduce the size of myocardial infarcts, and attenuate the effects of cardiac hypertrophy. While more research is needed to fully understand its effects and mechanisms of action, future results from clinical studies are expected to confirm the perspectives that so far suggest that nicotinamide riboside could become a valuable tool in the fight against cardiovascular disease through metabolism and mitochondrial dynamics.

### 4.4 Angiotensin- (1-9) and (1-7): Novel Cardioprotective Agents Modulating Mitochondrial Function

Angiotensin- [1-9] [Ang- (1-9)] and Angiotensin- (1-7) [Ang- (1-7)] are key components of the non-canonical renin-angiotensin system (RAS), which have been described as promising agents against cardiac disease [118]. Ang- (1-9) is generated from Ang-I by the proteolytic cleavage of ACE2 (Angiotensin Converting Enzyme 2). On the other side, Ang- (1-7) can be produced from Ang-II by ACE2, and from Ang-I, by several endopeptidases [119]. Ang- (1-9) has shown anti-hypertrophic and antihypertensive effects in several experimental models [120, 121], and additionally, has been described to regulate mitochondrial dynamics. Interestingly, Ang- (1-9) prevented norepinephrine (NE)-induced mitochondrial fragmentation and cardiomyocyte hypertrophy [122]. Ang- (1-9) increased DRP1-Ser637 phosphorylation (PKA-dependent phosphorylation site) [123], promoting mitochondrial fusion, through a novel mechanism involving miR-129-3p/PKIA and the positive regulation of PKA signaling [122]. This finding highlights the connection between mitochondrial dynamics dysregulation and cardiac hypertrophy [124].

On the other hand, (Ang- (1-7), has also shown cardioprotective actions. Using an *ex vivo* model of I/R, Ang- (1-7) enhanced the beneficial effects of ischemic preconditioning (IPC), reducing infarct size, apoptosis levels, and oxidative stress. Interestingly, Ang- (1-7) improved the effects of IPC over mitochondrial function, preserving mitochondrial respiration and membrane potential [125].

Ang- (1-9) and Ang- (1-7) are emerging as viable peptides in the prevention and treatment of heart disease, thanks to their influence on mitochondrial function. The mechanisms by which Ang- (1-9) exerts its beneficial effects on the heart are explained by the regulation of intracellular calcium and the inhibition of mitochondrial fission, crucial processes in cardiomyocyte hypertrophy, while Ang- (1-7) enhanced the effects of IPC on mitochondrial function, preserving mitochondrial respiration and membrane potential.

## CONCLUSION

In recent years, attention has increased toward the study of the role of mitochondrial dysfunction in the development and progression of cardiovascular diseases (CVD).

Although the pathophysiological complexity underlying CVD cannot be addressed with a single pharmacological approach designed to prevent or resolve mitochondrial damage and dysfunction, therefore, improving our understanding of mitochondria-dependent mechanisms in CVD may become a useful option to develop additional potential therapeutic targets for the treatment of these pathologies.

The effects of Metformin (MET) on mitochondrial apoptosis and fission suggest its potential positive effect in regulating excessive cardiac cell growth, characteristic of cardiac hypertrophy. MET also reduces the production of reactive oxygen species (ROS) and protects against the alteration of mitochondrial membrane potentials (MMP). This protective effect of metformin is mediated by the activation of AMPK and its influence on PGC-1α signaling.

On the other hand, one of the therapeutic strategies that appear on the horizon is Resveratrol (RES), which recent studies demonstrate its ability to significantly improve mitochondrial function in cardiomyocytes damaged by hypoxia/reoxygenation. Similarly, other studies have shown promising results with the pharmacological potential of Nicotinamide Riboside (NR). Oral administration of NR for 8 weeks was found to improve NAD+ deficiency and reverse hyperacetylation of enzymes such as very long-chain acyl-CoA dehydrogenase (VLCAD) in hearts with heart failure with preserved ejection fraction (HFpEF) in mice. This positive effect on NAD+ levels and mitochondrial function suggests that NR could have a therapeutic role in improving cardiac function in the context of HFpEF.

On the other hand, Ang- [(1-9)] and Ang- [(1-7)] peptides have shown cardioprotective effects in several experimental models and, in addition, have been described to regulate mitochondrial dynamics. Ang- [(1-9)] has been shown to prevent norepinephrine (NE)-induced mitochondrial fragmentation and cardiomyocyte hypertrophy, and using an *ex vivo* I/R model, Ang- [(1-7)] enhanced the beneficial effects of ischemic preconditioning (IPC), reducing infarct size, apoptosis levels, and oxidative stress.

Finally, the cardioprotective effects of drugs that preserve mitochondrial function in CVD are well documented based on several clinical trials, however, further efforts are required to find safe and effective drugs. Additionally, future research should focus on the identification and validation of specific mitochondrial biomarkers, which could serve not only as diagnostic and prognostic tools but also as indicators of therapeutic response. These biomarkers would be crucial for personalizing treatments and for early detection of mitochondrial dysfunction in cardiovascular diseases.

## Figures and Tables

**Table 1:** *In vitro*, *ex vivo,* and *in vivo* studies about the effects of MET, nicotinamide, and resveratrol on the regulation of mitochondrial function in cardiac diseases.

**Drug**	**Disease**	***In vitro* and** ***Ex vivo* Studies**	***In vivo* Studies**	**Dosage**	**Observation**
Metformin	Ischemia/reperfusion cardiac	-----	Male Rats Wistar	100, 200 mg / kg iv 2 mM on heart	Cardioprotectors effects during IR acute on rats [90].
Metformin	Cardiac hypertrophy	Fetal cell line derived from rat cardiomyocytes (H9c2)	Male Rats Sprague-Dawley	30 mg/kg / day SD rats 1 mM in H9c2 cells	Cardioprotective effects by attenuating mitochondrial fission, apoptosis, and arrhythmias, in addition to preserving left ventricular function [91].
Metformin	Retinal Isquemia/reperfusión	The cell line Rat R28	-----	25 μg por 4 h concentration of 0,2 mM	Protective effects against retinal ischemia/reperfusion injury through AMPK-mediated mitochondrial fusion [92].
Nicotinamide riboside	Heart failure with preserved ejection fraction	----	Rats C57BL/6N adult males	Oral supplement 400 mg/Kg/day for 8 weeks	Oral supplementation with NR improved mitochondrial function in HFpEF hearts [111].
Nicotinamide and resveratrol riboside	Cardiac ischemia/reperfusion	----	Male mice C57BL/6, 8 to 10 weeks old	NR and resveratrol microspheres, 100 mg NR/kg	Oral administration of [NR/RESms] protects the heart against cardiac I/R [115].
Nicotinamide riboside	Cardiac hypertrophy	----	Male mice C57BL/6J, 8 weeks old	Daily oral gavage 400 mg/kg/day for 8 weeks	NR could have a positive impact on cardiac hypertrophy by modulating the NLRP3-Sirtuin3-MnSOD inflammasome signaling pathway [116].
Resveratrol	Myocardial ischemia/reperfusion	Neonatal primary cardiomyocytes from Sprague-Dawley rats	-----	5 µM and 20 µM in incubation for 12 h.	Significant protective effect on the mitochondria of myocardial cells damaged by hypoxia/reoxygenation [101].
Resveratrol	Myocardial ischemia/reperfusion	Rat neonatal ventricular myocytes (NRVM) and H9c2 cells with expression of AMPK, SIRT1 or FOXO1	Adult male C57BL/6J mice weighing 20-25 g	Mice: intraperitoneal injection 30 mg/kg per day for 7 days. L. Cellular: 30 μM for 24 h	Decrease in the size of myocardial infarction, improves cardiac function and reduces oxidative stress caused by MI/IR [102].
Resveratrol	Coronary microembolization	-----	Sprague-Dawley mice	25 mg/kg and 50 mg/kg per day, by tube for 7 days	Protective effect of mitochondrial function and structural integrity in cardiac tissue subjected to stress [103]

**Table 2:** Clinical trials about the effects of MET, resveratrol, and nicotinamide on the regulation of mitochondrial function in heart diseases.

**Trial Name**	**Phase**	**Diseases**	**Treatment**	**Participants**	**Sponsor**
Effects of metformin treatment on myocardial efficiency in patients with heart failure (METRÓNOMO) (NCT02810132) [93]	2	Systolic heart failure	Drug: Metformin Drug: Placebo	36	Aarhus University Hospital
Cardioprotective and metabolic effects of metformin in patients with heart failure and diabetes (CARMET) (NCT01690091) [94]	2,3	Insulin resistance Chronic heart failure	Drug: Metformin hydrochloride (Siofor 1000 tbl, Berlín) Drug: placebo	40	Institute of Clinical and Experimental Medicine
Evaluation of the clinical efficacy of resveratrol in improving metabolic and skeletal muscle function in patients with heart failure (REV-HF) (NCT03525379) [104]	2	Chronic congestive heart failure	Drug: Resveratrol Drug: Placebo	13	Alberta University
Resveratrol improves cardiac function by moderating inflammatory processes in patients with systolic heart failure [105]	2-3	Systolic heart failure	Drug: Resveratrol Drug: Placebo	60	Pecs University
Nicotinamide Riboside in LVAD Recipients (PilotNR-LVAD) (NCT03727646) [112]	Early 1	Congestive heart failure Heart failure New York Heart Association Class IV Mitochondrial alteration	Dietary Supplement: Nicotinamide Riboside	10	Washington University
Mechanistic studies of nicotinamide riboside in human heart failure (NRII) (NCT04528004) [117]	Early 1	Systolic heart failure NYHA Class IV heart failure Metabolic alteration	Drug: Nicotinamide riboside Other: Placebo	40	Washington University

## LIST OF ABBREVIATIONS

ACE2: Angiotensin Converting Enzyme 2
ACS: Acyl-CoA Synthetase
AhR: Aryl Hydrocarbon Receptor
AMPK: AMP-Dependent Kinase
AT1R: Angiotensin Type 1 Receptor
BNP: Brain Natriuretic Peptide
CFs: Cardiac Fibroblasts
CMFs: Cardiac Myofibroblasts
COX: Cyclooxygenases
CVD: Cardiovascular Disease
DRP1: Dynamin-related Protein 1
EC: Endothelial Cells
ECG: Electrocardiogram
ECM: Extracellular Matrix
eNOS: Endothelial Nitric Oxide Synthase
ETC: Electron Transport Chain
FABP: Fatty Acid Binding Protein
FABP5: Fatty Acid Binding Protein 5
FB: Fibroblasts
FFAs: Free Fatty Acids
HF: Heart Failure
I/R: Isquemia/Reperfusion
IPC: Ischemic Preconditioning
LCFAs: long-chain Fatty Acids
LV: Left Ventricular
LVEF: LV Ejection Fraction
MCU: Mitochondrial Calcium Uniporter
MDA: Malondialdehyde
MET: Metformin
MMP: Mitochondrial Membrane Potential
mPTP: Mitochondrial Permeability Transition Pore
mTOR: Mammalian Target of Rapamycin
NAD+: Nicotinamide Adenine Dinucleotide
NAM: Nicotinamide
NE: Norepinephrine
NF-κB: Nuclear Factor Kappa B
NMN: Nicotinamide Mononucleotide
NR: Nicotinamide Riboside
NRF2: Nuclear Factor Erythroid 2 Related to Factor 2
OMM: Outer Mitochondrial Membrane
OPA1: Optic Atrophy 1
PBMCs: Peripheral Blood Mononuclear Cells
PDE: Phosphodiesterases
PERK/ATF4: Protein Kinase R Kinase/ Activating Transcription Factor 4
PI3K: Phosphoinositide 3-Kinase
PKA: Protein Kinase A
RAAS: Renin-Angiotensin-Aldosterone System
RES: Resveratrol
ROS: Reactive Oxygen Species
SIRT1: Sirtuin 1
SMC: Smooth Muscle Cells
SOD: Superoxide Dismutase
SOD2: Superoxide Dismutase 2
TCA: Tricarboxylic Acid
VEGF: Vascular Endothelial Growth Factor
VLCAD: Very Long-Chain Acyl-CoA Dehydrogenase
